# Editorial: Biofluid biomarkers for the diagnosis of neurodegenerative diseases: current status

**DOI:** 10.3389/fnins.2026.1985558

**Published:** 2026-09-21

**Authors:** Boris Decourt, Michelle M. Mielke, Paul Desmond Slowey, Marwan N. Sabbagh

**Affiliations:** 1 Department of Pharmacology and Neuroscience, School of Medicine, Texas Tech University Health Sciences Center, Lubbock, TX, United States; 2 Department of Epidemiology and Prevention, Wake Forest University School of Medicine, Winston-Salem, NC, United States; 3 Oasis Diagnostics Corporation, Vancouver, WA, United States; 4 Alzheimer's and Memory Disorders Division, Barrow Neurological Institute, Phoenix, AZ, United States

**Keywords:** biofluid, biomarker, clinical trial, context of use (COU), measurement platform, neurodegenerative diseases, tissue matrix

The incidence of neurodegenerative diseases (NDDs), including Alzheimer's disease, Parkinson's disease, Huntington's disease, and amyotrophic lateral sclerosis among others, continues to rise worldwide as the global population ages. Spanning high, medium, and low income countries altogether, the incidence of NDDs is expected to double by 2060 (Alzheimer's Association, 2026). This is accompanied by an increasing societal and familial burden as the current healthcare cost estimates for NDD patients range from $15,000 to $115,000 annually (Alzheimer's Association, 2026; Aamodt et al., 2024; Gokhale and Villa Zapata, 2025). These figures highlight the urgent need to develop novel, effective therapies for NDDs. But therapeutic progress can only advance if paired with disease-relevant biomarkers.

This Research Topic presents 11 articles that address numerous important considerations regarding the current status of biofluid biomarkers research in the management of NDDs. The first consideration is the disease of interest among NDDs. Ideally, a biomarker will provide information on the type of NDD a patient may be suffering, eventually identifying mixed pathology as well, and the actual disease stage. The articles by Wang et al.; Gleerup et al.; Zhang et al.; and Gragera-Martinez et al. illustrate this point. The second consideration is the context of use, with options ranging from screening to diagnosis, prognosis, theragnostic applications, and monitoring treatment effectiveness, as investigated by Gragera-Martinez et al. and Chen et al.. These first two parameters guide the next two considerations, which are the type of patient administered a biofluid marker test and the determination of cut-off values. For example, screening biomarkers could be deployed in the general population, while diagnostic biomarkers would focus on symptomatic patients in an effort to determine etiology, as done by Oh et al.. Importantly, asymptomatic patients may return positive biomarker tests because the pathology of many NDDs begin years before symptom onset, as reported by Zhang et al.. Disease is diagnosed only when a biomarker test reaches the pre-determined cut-off values. However, cut-offs may depend on demographics (e.g., sex, ancestry, and ethnicity), medications used for the treatment of comorbidities (Brum et al., 2024), or the measurement platform used to carry out a given biomarker test (Anastasi et al., 2025). These examples emphasize the need for conducting large scale studies across diverse populations that investigate several variables in parallel with sufficient statistical power for each variable.

In addition to the parameters above, one must also consider biological and methodological criteria when developing or conducting biofluid marker measurements. First, there is the choice of matrix used, which include cerebrospinal fluid (CSF), blood, saliva (e.g., Gleerup et al. and Licatini et al.), urine (e.g., Chen et al.), tears, and vitreous humor. The choice depends on the abundance of a chosen biomarker (both in physiological and disease states) in the specific biofluid, the risk associated with the collection method (e.g., CSF is considered more invasive than blood and saliva collection), and the platform(s) available for biomarker measurement (e.g., Tortosa-Carreres et al.). Some biofluids may need pre-processing to isolate fractions/matrices that contain the biomarker of interest, for example centrifugation of CSF (e.g., Türkegün Sengül and Nemutlu Samur), blood, and saliva to separate cells and extracellular vesicles (EVs) from biofluids; or the preparation of blood serum vs. plasma. Further, several types of biomarkers can be identified in each matrix, including fragmented DNA, micro RNAs, circular RNAs, non-coding RNAs (e.g., Yadollahi Khales et al.), messenger RNAs, native proteins, post-translationally modified proteins, lipids, and carbohydrates (Hodgins et al., 2026). The type of biomarker also determines the method used to conduct measurements. For example, DNA and RNA molecules are often measured via PCR methods, while native and modified proteins can be analyzed either by singleplex or multiplex ELISA methods, mass spectrometry, enzymatic activity assays, immunohistochemistry, immunocytochemistry, cell sorting, or oligomerization/seed amplification assays. Each method and measurement platform displays unique biophysical parameters making them more or less relevant to the measurement of a given biomarker: this includes lower and higher limits of detection, possible interferences due to matrix effects, binding affinity of proprietary antibodies, and pre-processing methods.

In aggregate, lessons learned #1: multiple parameters need to be considered in the development and use of biomarkers for NDDs. In recent years, it has become increasingly apparent that panels of multimodal markers hold stronger potential than single markers in either context of use (e.g., Wang et al.), which has stimulated many business opportunities to identify highly pertinent biomarker panels for the improved diagnosis of NDDs. This is highlighted by the approval by the FDA in recent years of CSF- and blood-based biomarkers for the diagnosis of Alzheimer's disease. Consequently, more physicians request biofluid-based biomarkers for patients presenting with memory complaints and integrate the test results in their stepwise algorithms used to develop personalized treatment plans. For example, the two currently FDA-approved immunotherapies for the treatment of Alzheimer's require administration at early stage of the disease. Thus, physicians need to first diagnose patients with Alzheimer's disease, then ascertain whether the disease is early stage. In the future, physicians may need to consider treatment initiation for biomarker-positive asymptomatic patients. In such a case scenario, it will be important to determine whether a positive test result is both associated with neuropathology and prognostic of future conversion to symptoms.

Lessons learned #2: this Research Topic of articles illustrate the current state of research on biofluid biomarkers for NDDs. While some biomarkers are now used in daily clinical practice for Alzheimer's management, there remain numerous areas of investigation to develop novel platforms and biomarkers to cover the full spectrum of NDDs and determine their context of use as well as their longitudinal trajectory both in disease and non-disease states. Since most NDDs have no cure at present, the most sought-after approach is the modification of lifestyles to either slow down or reverse early disease. Such paradigms may require the measurement of biomarkers among patients suffering NDDs, and also their family members who are often exposed to similar lifestyle and microenvironment (e.g., Chen et al.).

Lessons learned #3: research on biofluid biomarkers for NDDs is still at its infancy and will require numerous additional, carefully designed studies to reach a stage of clinical development where multiple NDDs can be screened or diagnosed using multimodal panels of markers from a single biofluid collection. Furthermore, the development of biofluid biomarkers is an opportunity to identify cost-effective makers that can be used in patient stratification strategies and to assess treatment efficacy in clinical trials.

## References

[B1] Aamodt W. W. Sun C. Dahodwala N. Elser H. Schneider A. L. C. Farrar J. T. . (2024). End-of-life health care service use and cost among medicare decedents with neurodegenerative diseases. Neurology 103:e209925. doi: 10.1212/WNL.0000000000209925 39393030 PMC11469682

[B2] Alzheimer's Association (2026). 2026 Alzheimer's disease facts and figures. Alzheimers Dement. 22:e71345. doi: 10.1002/alz.71345

[B3] Anastasi F. Fernandez-Lebrero A. Ashton N. J. Ortiz-Romero P. Torres-Torronteras J. Gonzalez-Escalante A. . (2025). A head-to-head comparison of plasma biomarkers to detect Alzheimer's disease in a memory clinic. Alzheimers Dement. 21:e14609. doi: 10.1101/2024.10.26.24316176 PMC11852974 39998475

[B4] Brum W. S. Docherty K. F. Ashton N. J. Zetterberg H. Hansson O. McMurray J. J. V. . (2024). Effect of neprilysin inhibition on Alzheimer disease plasma biomarkers: a secondary analysis of a randomized clinical trial. JAMA Neurol. 81, 197–200. doi: 10.1001/jamaneurol.2023.4719 PMC10728797 38109077

[B5] Gokhale P. Villa Zapata L. (2025). Economic burden of Huntington's disease: a systematic review. J. Huntingtons Dis. 14, 30–42. doi: 10.1177/18796397251319209 39973389

[B6] Hodgins M. L. Maxwell S. P. Howlett S. E. Rockwood K. (2026). Blood biomarkers of frailty and cognition: a scoping review. Neurobiol Aging 161, 14–30. doi: 10.1016/j.neurobiolaging.2026.01.003 41544446

